# Effect of bromocriptine alginate nanocomposite (BANC) on a transgenic *Drosophila* model of Parkinson's disease

**DOI:** 10.1242/dmm.022145

**Published:** 2016-01-01

**Authors:** Yasir Hasan Siddique, Wasi Khan, Ambreen Fatima, Smita Jyoti, Saba Khanam, Falaq Naz, Rahul, Fahad Ali, Braj Raj Singh, Alim Hussain Naqvi

**Affiliations:** 1 Drosophila Transgenic Laboratory, Section of Genetics, Department of Zoology, Faculty of Life Sciences, Aligarh Muslim University, Aligarh, 202002, Uttar Pradesh, India; 2 Centre of Excellence in Materials Sciences (Nanomaterials), Department of Applied Physics, Z. H. College of Engineering & Technology, Aligarh Muslim University, Aligarh, 202002, Uttar Pradesh, India

**Keywords:** Bromocriptine, Nanocomposite, Parkinson's disease

## Abstract

The effect of bromocriptine, a dopamine agonist, administered in the form of bromocriptine alginate nanocomposite (BANC) was studied on Parkinson's disease (PD) model flies. The synthesized BANC was subject to characterization and, at a final concentration of 0.5, 1.0 and 1.5 µM, was mixed in diet. The PD flies were allowed to feed on it for 24 days. A significant dose-dependent delay in the loss of climbing activity and activity pattern was observed in PD flies exposed to 0.5, 1.0 and 1.5 µM BANC. The PD flies exposed to BANC also showed a significant reduction in lipid peroxidation and glutathione-S-transferase activity, and an increase in glutathione content. However, no gross morphological changes were observed in the brains of PD flies compared with controls. The results suggest that BANC is effective in reducing the PD symptoms in these transgenic flies.

## Abstract (precis)

**Summary:** The results suggest that the bromocriptine alginate nanocomposite is potent in reducing the symptoms of Parkinson's disease in a transgenic fly model of the disease.

## INTRODUCTION

Dopamine agonists can show neuroprotective properties via several mechanisms, such as the levodopa-sparing effect, decreased dopamine turnover, antioxidant activity or inhibition of the subthalamic nucleus (Szczudlik and Rudzinska, 2007). Bromocriptine (2-bromo-α-ergocryptine methanesulfonate) is a semi-synthetic ergot alkaloid used for the treatment of diseases caused by hyperprolactinemia and as a dopamine agonist in some neurological disorders (Kitamura et al., 2003). It is absorbed through the gastrointestinal tract but has low bioavailability because of the hepatic first-pass effect (Cedarbaum, 1987). Dopamine agonist therapy can lead to impulse control disorder via dopamine D3 receptors owing to its high affinity for the dopamine D2 and D3 receptors (Seeman, 2015). The dopamine agonists, in addition to anti-Parkinson's effects, also have side effects in the form of gastrointestinal effects, headache and dizziness (Cedarbaum, 1987), valve regurgitation (Rasmussen et al., 2011), valvular heart disease (Tan et al., 2009) and sleepiness (Micallef et al., 2008). Bromocriptine not only inhibits the release of glutamate by reversing the glutamate GLT1 transporter (Shirasaki et al., 2010) but also worsens the psychotic symptoms (Boyd, 1995). The induction of pulmonary fibrosis has been reported at the higher doses of bromocriptine (Todman et al., 1990). It has now become more important to find convenient, effective and safe ways for the use of therapeutic drugs for central nervous system (CNS) disorders. The diagnosis and treatment of CNS diseases have been greatly facilitated by the advancement in biomedical and pharmaceutical applications of nanotechnology (Yang, 2010). Nanoparticles are useful not only in maintaining drug levels in a therapeutically desirable range but they also increase the half-life, solubility, stability and permeability of drugs (Yang, 2010). In the present study, bromocriptine alginate nanocomposite (BANC) was synthesized, characterized and studied in a transgenic *Drosophila* model of Parkinson's disease (PD).

## RESULTS AND DISCUSSION

**Fig. 1.**
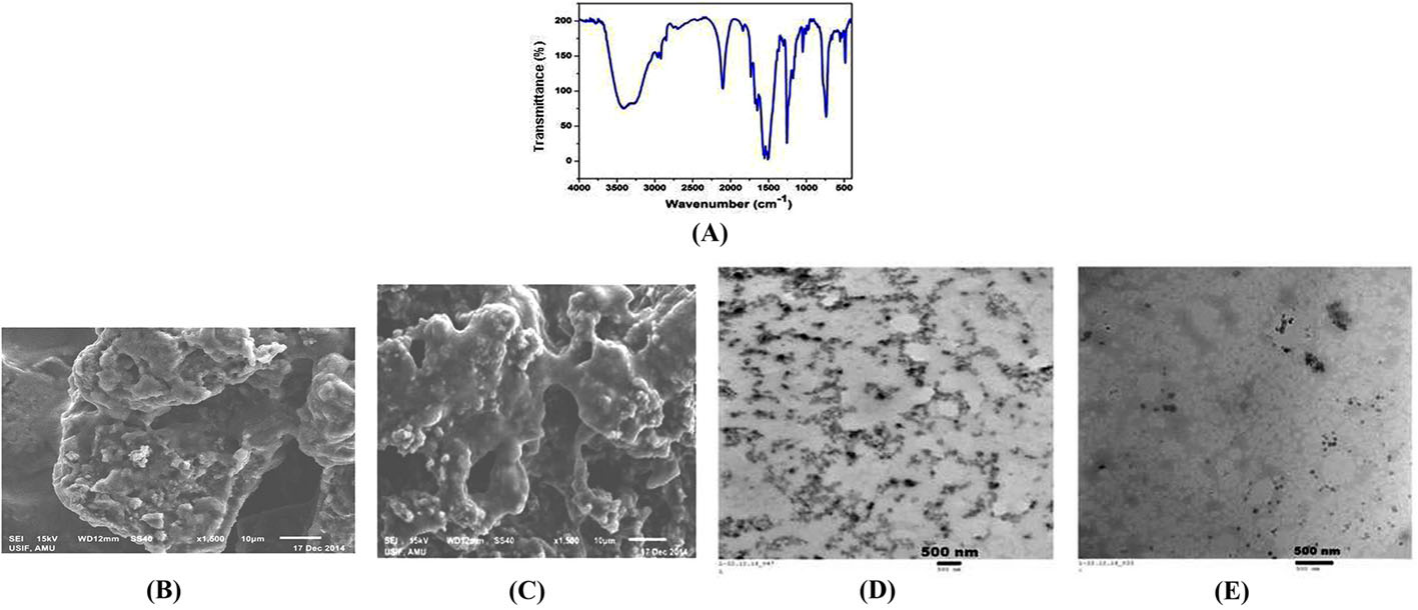
**Characterization of bromocriptine alginate nanocomposite (BANC).** FTIR spectrum of BANC (A). Scanning electron microscopy images (B,C) and transmission electron micrographs (D,E) of bromocriptine and its nanocomposite with alginate.

Information about the chemical bonding in a sample can be determined by Fourier transform infrared (FTIR) spectroscopy. To confirm the purity and formation of BANC, infrared spectroscopy was used in the wave number range 4000-400 cm^−1^ (Fig. 1A). The band at 3500 cm^−1^ was assigned to an -OH bending of hydroxide phase; 2300 cm^−1^, 1500 cm^−1^ and 1200 cm^−1^ were attributed to the stretching vibrations of the -CO, -C=O and -C-O-C bonds, respectively. The bands at 700 cm^−1^ are due to the -CH bending and might have appeared owing to the gases surrounding the sample, confirming bond formation between bromocriptine and alginate.

The scanning electron microscopy (SEM) images of bromocriptine and its nanocomposite with alginate are shown in Fig. 1B,C, respectively. The porous structures are seen clearly in SEM images. The nanostructure size is narrow in distribution in the composite sample as compared to pure drug and, because of the morphology, the surface is nearly in a uniform agglomeration. These images illustrate the presence of large spherical aggregates composed of smaller individual nanostructures of different sizes.

Supplementary observations on the morphology and nanostructure formation can be established through the transmission electron microscopy (TEM) measurement. Fig. 1D,E show the TEM micrographs for pure drug and BANC, respectively, showing that the particles are almost spherical in shape with particle size distribution between 30 and 40 nm. These images clearly show spherical particles with a highly agglomerated nature. The average particle size was estimated by considering the minimum and maximum diameter of a large number of nanoparticles (Khan et al., 2010). It is clear from Fig. 1E that the average size of the particles reduces to 20 nm with alginate. The distribution of particles is quite uniform throughout the BANC sample.

**Fig. 2.**
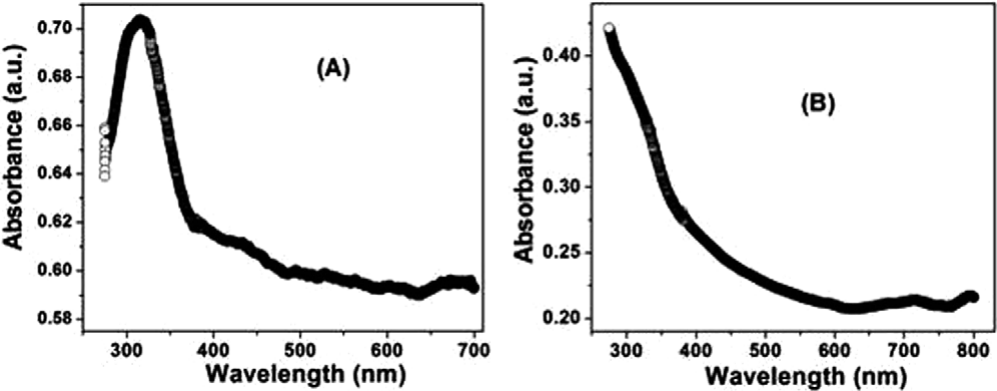
**Ultraviolet-visible absorption spectroscopy performed for bromocriptine and its nanocomposite with alginate.** (A) Bromocriptine. (B) BANC.

The optical properties of nanostructures can be studied by UV-visible absorption spectroscopy. The dopant changes the band gap of materials, resulting in different absorbance values. The absorbance can also depend on factors such as particle size, oxygen deficiency and defects in grain structure (Hassan et al., 2014). The optical absorption of bromocriptine and BANC was investigated in the wavelength range 200-800 nm, as shown in Fig. 2A,B. It is clear from the spectrum that the strong absorbance is found for the pure drug at a wavelength of nearly 320 nm, whereas a low absorbance is observed in the visible region.

**Fig. 3.**
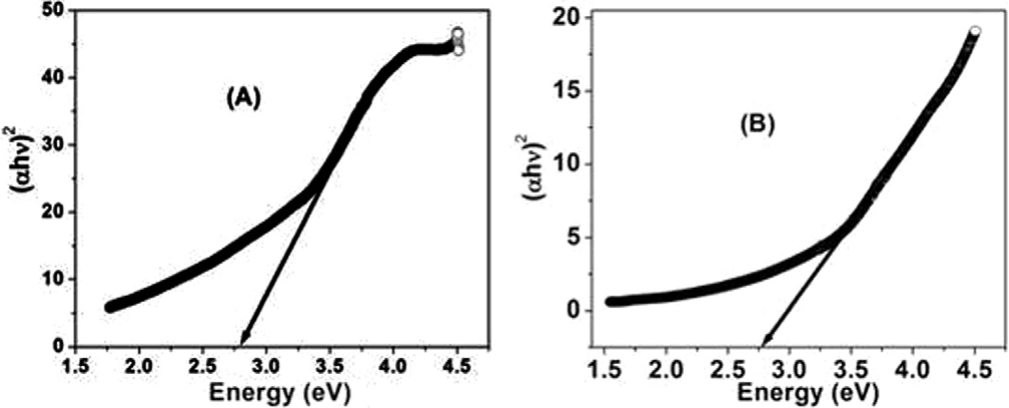
**Band** **gap plots for bromocriptine and its nanocomposite with alginate.** (A) Bromocriptine. (B) BANC.

BANC did not show a significant shift towards higher wavelengths, which means that the band gap decreases slightly owing to the defects inside the materials and crystallite size of the drug. The energy band gap (*E_g_*) was calculated using Tauc's relation, α*hν*=*B*(*hν*-*E_g_*)*^n^*, where α is the absorption coefficient, *n*=1/2 or 2, for direct or for indirect allowed transitions respectively, and *B* is the characteristic parameter for respective transitions, regardless of photon energy *hν*. The band gap was found to be nearly 2.76 eV for both samples, indicating that the addition of alginate does not affect their characteristics, whereas the absorption range and order increases. Band gap graphs were plotted in the linear region near the onset of (α*hν*)^2^ versus *hν*, which is shown in Fig. 3A,B for both samples.

**Fig. 4.**
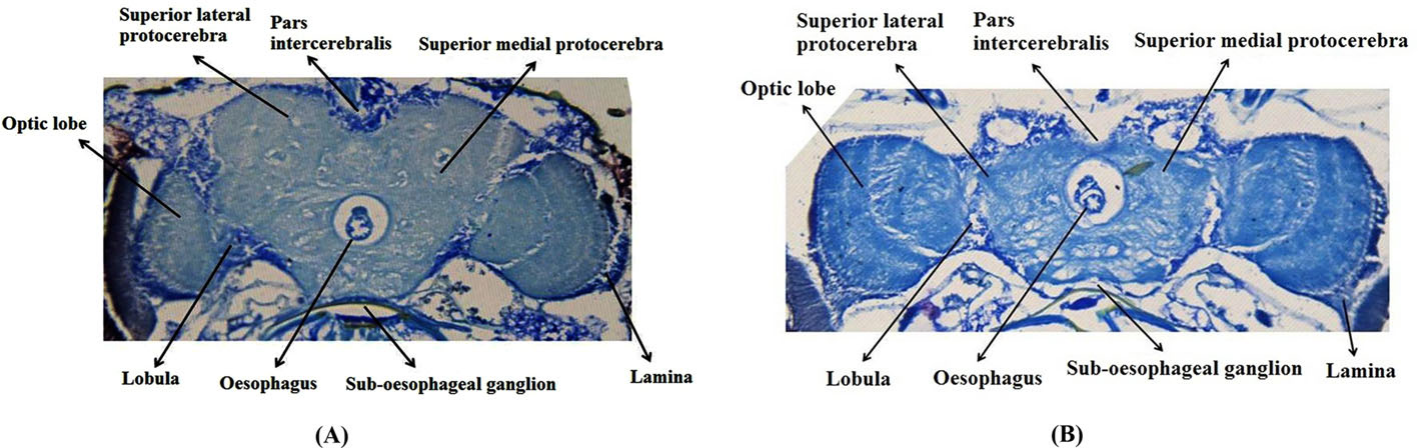
**Microscopic illustrations of the brain of *Drosophila melanogaster* stained by toludine blue.** (A) Control fly. (B) PD fly.

**Fig. 5.**
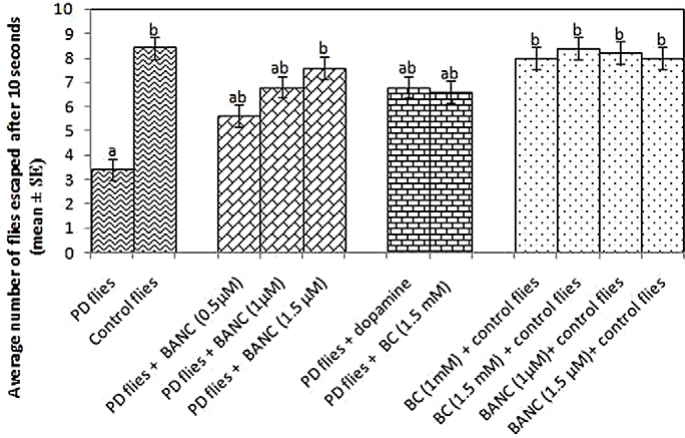
**Effect of BANC on climbing ability.** The flies (ten flies per treatment; five replicates per group) were allowed to feed on the diet supplemented with BANC for 24 days and then assayed for climbing ability. The values are the mean of five assays. ^a^Significant with respect to control, *P*<0.05; ^b^significant with respect to PD model flies; BANC, bromocriptine alginate nanocomposite; BC, bromocriptine; dopamine: 10^−3^ M.

The histological sections of the control and PD flies showed no gross morphological changes in the brain (Fig. 4A,B). The results obtained for the climbing ability are shown in Fig. 5. A significant dose-dependent delay in the loss of climbing ability of PD flies exposed to 0.5, 1.0 and 1.5 µM of BANC was observed (Fig. 5; *P*<0.05). A significant delay in the loss of climbing ability was also observed in PD flies exposed to 10^−3^ M of dopamine and 1 mM of bromocriptine, compared to untreated PD flies (Fig. 5; *P*<0.05). The studied doses of BANC and bromocriptine (alone) did not show any positive or negative effects on the climbing ability of control flies (Fig. 5; *P*<0.05).

The results obtained for the activity pattern are shown in Figs S1 to S11.The average activity pattern shows a dose-dependent improvement in the average activity of BANC-treated PD flies as compared to untreated PD flies. The exposure of control flies to 0.5, 1 and 1.5 µM of BANC did not result in any change in the activity pattern of the flies (Figs S6-S8). The flies exposed to 10^−3^ M dopamine and 1.5 mM of bromocriptine also showed an improvement in the activity pattern of PD flies (Figs S9-S10).

**Fig. 6.**
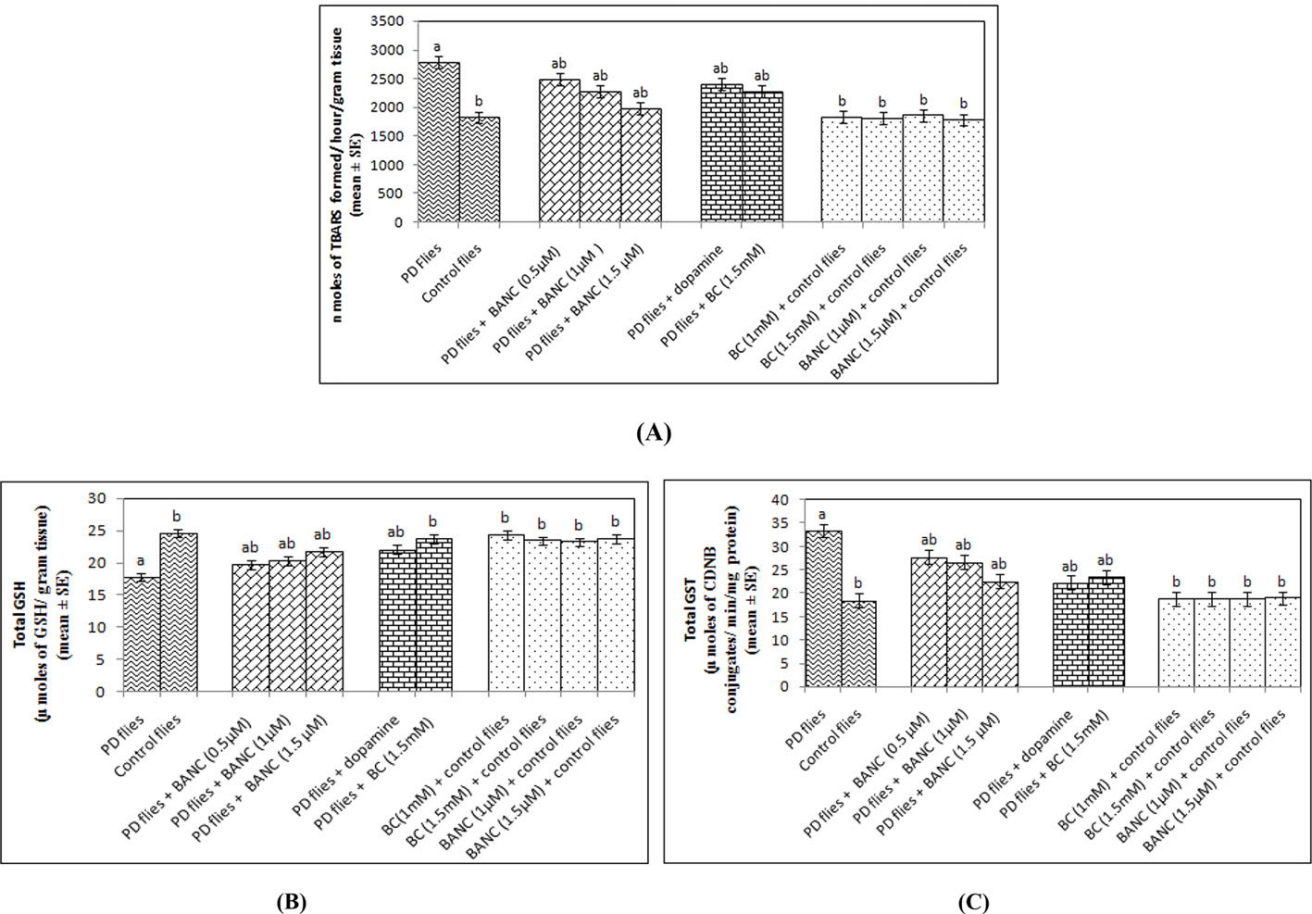
**Effect of BANC on lipid peroxidation, GSH content and GST activity in the brains of flies.** Lipid peroxidation (A), GSH content (B) and GST activity (C) in the brains of flies are shown. The flies were allowed to feed on the diet supplemented with BANC for 24 days and then assayed for lipid peroxidation. The values are the mean of five assays. ^a^Significant with respect to control, *P*<0.05; ^b^significant with respect to PD model flies. BANC, bromocriptine alginate nanocomposite; BC, bromocriptine; CDNB, 2,4-dinitrochlorobenzene; dopamine, 10^−3^ M; GSH, glutathione; GST, glutathione-S-transferase; TBARS, thiobarbituric acid reactive species.

The results obtained for lipid peroxidation (LPO) are shown in Fig. 6A. A significant 1.52-fold increase was observed in PD flies as compared to control flies (Fig. 6A; *P*<0.05). The PD flies exposed to 0.5, 1 and 1.5 µM of BANC showed a dose-dependent 1.12-, 1.22- and 1.41-fold decrease in LPO in the brains of PD model flies as compared to untreated PD flies (Fig. 6A; *P*<0.05). The control flies exposed to 0.5, 1 and 1.5 µM of BANC did not show any significant increase in LPO as compared to untreated control flies (Fig. 6A; *P*<0.05). The flies exposed to 10^−3^ M of dopamine or 1.5 mM of bromocriptine showed a 1.14- and 1.20-fold decrease, respectively, in LPO as compared to untreated PD flies (Fig. 6A; *P*<0.05). The results obtained for total glutathione (GSH) content is shown in Fig. 6B. A significant 1.38-fold decrease in the GSH content was observed in PD flies as compared to control flies (Fig. 6B; *P*<0.05). The exposure of 0.5, 1 and 1.5 µM of BANC to PD flies showed a dose-dependent 1.10-, 1.14- and 1.22-fold increase in GSH content as compared to PD flies alone (Fig. 6B; *P*<0.05). However, the increase was less compared with the control group. The control flies exposed to 1 and 1.5 µM of BANC did not show any significant increase or decrease in GSH content (Fig. 6B; *P*<0.05). The PD flies exposed to 10^−3^ M of dopamine or 1.5 mM of bromocriptine showed a 1.24- and 1.33-fold increase in GSH content, respectively (Fig. 6B; *P*<0.05). The results obtained for the glutathione-S-transferase (GST) activity is shown in Fig. 6C. A significant 1.80-fold increase in GST activities was observed in PD flies as compared to control flies (Fig. 6C; *P*<0.05). The exposure of PD flies to 0.5, 1 and 1.5 µM of BANC resulted in a dose-dependent 1.20-, 1.25- and 1.47-fold significant decrease as compared to untreated PD flies (Fig. 6C; *P*<0.05). The control flies exposed to 1 and 1.5 µM of BANC did not show any significant increase in GST activity (Fig. 6C; *P*<0.05). The PD flies exposed to 10^−3^ M of dopamine or 1 mM of bromocriptine showed a 1.50- and 1.42-fold decrease, respectively, in GST activity as compared to untreated PD flies (Fig. 6C; *P*<0.05).

The results of our present study reveal that BANC not only delays the loss of climbing activity but also reduces the oxidative stress in PD model flies. PD is a movement disorder characterized by the loss of dopaminergic neurons in the substantia nigra (Feany and Bender, 2000). The abnormal expression of alpha synuclein (αS) leads to the formation of Lewy bodies. As a result, the dopaminergic neurons are lost and the flies exhibit behavioural abnormality (Feany and Bender, 2000; Pendleton et al., 2002; Lessing and Bonini, 2009). The selective loss of dopaminergic neurons in the substantia nigra pars compacta, due to the formation of Lewy bodies, results in the decrease of brain dopamine content, ultimately leading to the motor defects (Muñoz-Soriano and Paricio, 2011). Oxidative stress an important factor in PD and occurs as a result of the formation of Lewy bodies (Butterfield and Kanski, 2001; Giasson et al., 2000; Sharon et al., 2003). In our present study with a transgenic *Drosophila* model of PD in which the flies express human αS, an increase in LPO and GST activity were observed in PD flies as compared to controls. The flies exposed to BANC showed a dose-dependent decrease in LPO and GST activities. This decrease was more effective in BANC-treated PD flies as compared to the PD flies exposed to bromocriptine alone. Bromocriptine is a potent free-radical scavenger (Yoshikawa, 1993; Ogawa et al., 1993; Yoshikawa et al., 1994) and has been reported to completely inhibit LPO in rat brain (Yoshikawa, 1993; Yoshikawa et al., 1994). In some reports, bromocriptine has been reported to stimulate antioxidant defence mechanisms, thereby serving as a neuroprotectant (Lim et al., 2009; Avila-costa et al., 2005). Oxidative damage as a result of oxidative stress alters many biological molecules, such as lipids, proteins and nucleic acids (Watson et al., 1984). Bromocriptine-mediated protection against ischemia-induced neuronal damage has been reported, which could result from the possible preservation of superoxide dismutases (SODs) by bromocriptine (Liu et al., 1995). Bromocriptine inhibits LPO in a dose-dependent manner in rat brain (Liu et al., 1995). A significant reduction in the GSH content was observed in flies expressing human αS, compared with controls. The exposure of PD flies to BANC prevented the loss of GSH content in a dose-dependent manner. This effect might also be attributed to the free-radical-scavenging property of bromocriptine. In various studies on humans, treatment with higher doses of bromocriptine alone results in an improvement of the motor complications (Montastruc et al., 1994; Lees and Stern, 1981; Hely et al., 1994). In our present study, the PD flies exposed to BANC showed a dose-dependent delay in the loss of climbing ability and activity pattern. This delay in the loss of climbing ability as well as in activity pattern was more effective in PD flies exposed to BANC compared to PD flies exposed to bromocriptine alone. The side effects/risks of the drug at higher doses cannot be completely eliminated (Rektorová et al., 2003). With the advent of nanotechnology, the researchers have attempted an innovative drug delivery system to prolong the half-life of bromocriptine (Esposito et al., 2008). Our study on PD model flies showed that BANC was effective in reducing PD symptoms. As far as *Drosophila* as a test model is concerned, not only do 75% of the disease-related genes in humans have functional orthologs, but also 80-90% similarity in the conserved domains have been reported between fly and mammals (Celotto and Palladino, 2005; Reiter et al., 2001). Owing to ethical reasons, *Drosophila* has been given preference in research and testing as an alternative to mammals (Mukhopadhyay et al., 2003). In this context, *Drosophila* has an advantage in the initial discovery process, regardless of raw throughput. The post-screening costs are significantly reduced by using a living organism like *Drosophila* for the primary screening of various substances (Pandey and Nichols, 2011). With the advent of new techniques and implementation of nanotechnology there has been a revolution for brain-specific drug delivery, imaging and diagnosis. For the treatment of CNS diseases, new nanostructured carriers of high drug-loading capacity and brain-targeting ability are needed to be explored (Siddique et al., 2013; Yang, 2010).

The results obtained in our present study showed that, by creating nanocomposites, the dose of the drug can be reduced to get the desired effects, thereby also reducing the side effects/risk of the drugs.

## MATERIALS AND METHODS

### Synthesis of alginate/bromocriptine (2-bromo-α-ergocryptine methanesulfonate) nanocomposite (BANC)

To prepare BANC, 100 mg of bromocriptine (Sigma, USA) was dissolved in 5 ml ethanol and this solution was added drop-wise into the warm 0.10% (100 ml) aqueous solution of sodium alginate (Ottokemi, India). The mixture was then ultra-sonicated at 100 W with 30 kHz frequency for 10 min and heated at 50°C for 1 h. The obtained BANC mixture was then air-dried. The dried BANC powder was stored in amber colour vials at room temperature under dry and dark conditions.

### Characterization methods

To ensure the formation and morphology of BANC, a scanning electron microscope (JEOL JSM-6510LV) was used at 1500× magnification and operated at 15 kV. Transmission electron microscopy (TEM) images of pure (bromocriptine) and the prepared composite were captured with JEOL (JEM-2010). A transmission electron microscope was used to estimate the average size of the nanostructures. Fourier transform infrared (FTIR) spectra of the samples were recorded in the range of 400-4000 cm^−1^ by Perkin Elmer spectrometer (Model: Spectra Two) using KBr as a reference material in the form of pellets of 13 mm diameter and 1 mm thickness. Optical properties of the samples were investigated by the UV-visible spectrophotometer (Perkin Elmer-Lambda 35) in the 200-800 nm range.

### *Drosophila* culture and crosses

The flies were cultured on standard *Drosophila* food containing agar, corn meal, sugar and yeast at 25°C (Siddique et al., 2012). Crosses were set up using six virgin females of UAS-Hsap/SNCA.F5B and mated to three males of GAL4-elav. The progeny expressed human αS in the neurons and the flies were referred as PD flies (Feany and Bender, 2000). The PD flies were also exposed separately to different doses of BANC mixed in the culture medium. BANC was added in the medium at final concentrations of 0.5, 1.0 and 1.5 µM. As a negative control, the PD flies were allowed to feed on the diet supplemented with 10^−3^ M of dopamine and 1.5 mM of bromocriptine. The control flies (UAS-Hsap/SNCA.F) were also exposed to the selected doses of BANC to see any negative effects. All the tests were performed after 24 days of exposure.

### Histological evaluation of the *Drosophila* brain

The fly heads were removed and kept in 10% buffered neutral formalin for 24 h. Then, the fixed heads from control and PD flies were embedded in paraffin and processed for light microscopy by staining individual sections with toludine blue (Palladino et al., 2000).

### *Drosophila* climbing assay

The climbing assay was performed according to Pendleton et al. (2002). Ten flies were placed in each empty glass vial. After the acclimatization for 10 min at room temperature, every group was assayed at random to a total of ten trials for each. The number of flies above the mark of the vial was counted after 10 s of climbing and repeated ten times to get the mean number.

### *Drosophila* activity pattern analysis

From the 12th day the activity of flies (males) in all treated groups was analyzed by using Drosophila Activity Monitor (TriTek, USA). The activity was recorded every hour for a total of 311 h and the data was analyzed by Actogram J software. The results were presented as chi-square periodogram (Chiu et al., 2010; Rosato and Kyriacou, 2006).

### Estimation of glutathione (GSH) content

GSH content was estimated colorimetrically using Ellman's reagent (DTNB) according to the procedure described by Jollow et al. (1974). The supernatant was precipitated with 4% sulphosalicyclic acid (4%) in the ratio of 1:1. The samples were kept at 4°C for 1 h and then subjected to centrifugation at 5000 rpm (4000 ***g***) for 10 min at 4°C. The assay mixture consisted of 550 µl of 0.1 M phosphate buffer, 100 µl of supernatant and 100 µl of DTNB. The optical density (OD) was read at 412 nm and the results were expressed as µM of GSH/gram tissue.

### Estimation of glutathione-S-transferase (GST) activity

GST activity was determined by the method of Habig et al. (1974). The reaction mixture consists of 500 µl of 0.1 M phosphate buffer, 150 µl of 10 mM 2,4-dinitrochlorobenzene (CDNB), 200 µl of 10 mM reduced GSH and 50 µl of supernatant. The OD was taken at 340 nm and the enzyme activity was expressed as µM of CDNB conjugates/min/mg protein.

### Lipid peroxidation (LPO) assay

LPO was measured according to the method described by Ohkawa et al. (1979). The reaction mixture consisted of 5 µl of 10 mM butyl-hydroxy toluene (BHT), 200 µl of 0.67% thiobarbituric acid, 600 µl of 1% O-phosphoric acid, 105 µl of distilled water and 90 µl of supernatant. The resultant mixture was incubated at 90°C for 45 min and the OD was measured at 535 nm. The results were expressed as µM of thiobarbituric acid reactive species (TBARS) formed/h/gram tissue.

### Statistical analysis

The statistical analyses were done using Statistica Soft Inc. software. Student's *t*-test was applied to observe the significant difference between treatments and controls.
